# Epigenetic and Genotoxic Mechanisms of PFAS-Induced Neurotoxicity: A Molecular and Transgenerational Perspective

**DOI:** 10.3390/toxics13080629

**Published:** 2025-07-26

**Authors:** Narimane Kebieche, Seungae Yim, Claude Lambert, Rachid Soulimani

**Affiliations:** 1 LCOMS/Neurotoxicology and Bioactivity, University of Lorraine, 57070 Metz, France; 2 Luxembourg Centre for Systems Biomedicine, L-4367 Belvaux, Luxembourg; seungae.yim@uni.lu; 3 Immunology Lab, University Hospital Saint-Etienne, 42055 Saint-Etienne, France; claude.lambert@chu-st-etienne.fr

**Keywords:** per- and polyfluoroalkyl substances, neurotoxicity, histone, ncRNA, DNA methylation, histone acetylation, transgenerational

## Abstract

Per- and polyfluoroalkyl substances (PFAS) are persistent environmental pollutants that continue to raise concern owing to their ability to accumulate in living organisms. In recent years, a growing body of research has shown that PFAS can exert their toxicity through disruption of both DNA integrity and epigenetic regulation. This includes changes in DNA methylation patterns, histone modifications, chromatin remodeling, and interference with DNA repair mechanisms. These molecular-level alterations can impair transcriptional regulation and cellular homeostasis, contributing to genomic instability and long-term biological dysfunction. In neural systems, PFAS exposure appears particularly concerning. It affects key regulators of neurodevelopment, such as BDNF, synaptic plasticity genes, and inflammatory mediators. Importantly, epigenetic dysregulation extends to non-coding RNAs (ncRNAs), including microRNAs (miRNAs) and long non-coding RNAs (lncRNAs), which mediate post-transcriptional silencing and chromatin remodeling. Although direct evidence of transgenerational neurotoxicity is still emerging, animal studies provide compelling hints. Persistent changes in germline epigenetic profiles and transcriptomic alterations suggest that developmental reprogramming might be heritable by future generations. Additionally, PFAS modulate nuclear receptor signaling (e.g., PPARγ), further linking environmental cues to chromatin-level gene regulation. Altogether, these findings underscore a mechanistic framework in which PFAS disrupt neural development and cognitive function via conserved epigenetic and genotoxic mechanisms. Understanding how these upstream alterations affect long-term neurodevelopmental and neurobehavioral outcomes is critical for improving risk assessment and guiding future interventions. This review underscores the need for integrative research on PFAS-induced chromatin disruptions, particularly across developmental stages, and their potential to impact future generations.

## 1. Introduction

Per- and polyfluoroalkyl substances (PFAS) are a class of synthetic chemicals increasingly detected in the environment and human tissues, raising growing concern about their long-term health effects, particularly when exposure occurs during vulnerable developmental windows [1,2,3]. Beyond their persistence and bioaccumulation, PFAS exert toxicity through a range of molecular mechanisms that go beyond classical toxicokinetic models. Recent studies highlight that PFAS can disrupt both genomic and epigenomic integrity. This includes disruption of DNA methylation, histone post-translational modifications, chromatin remodeling, and DNA repair processes [1,4,5,6]. These effects are further amplified by PFAS-mediated modulation of nuclear receptor signaling [7,8] and the dysregulation of non-coding RNAs, such as microRNAs and long non-coding RNAs [9,10,11], which together contribute to aberrant transcriptional activity across multiple tissues, including the brain.

Importantly, these effects are not only immediate or reversible. An expanding body of research suggests that PFAS exposure may induce persistent, and in some cases transgenerational, reprogramming of gene expression via germline epigenetics and chromatin alterations [12,13,14]. Such findings raise important concerns about the lasting and heritable consequences of early-life exposure. In this paper, we synthesize current insights into the genotoxic and epigenetic mechanisms through which PFAS influence cellular function, developmental programming, and neurotoxicity. Particular emphasis is placed on mechanisms affecting neural gene regulation—including the suppression of neurotrophic factors like BDNF, impaired synaptogenesis, and chronic neuroinflammatory response—as well as systemic disruption that may converge on altered neurodevelopmental outcomes. Altogether, these findings underscore the role of chromatin-level dysregulation as a central mechanism of PFAS toxicity, with implications that extend across molecular, cellular, and generational scales.

## 2. PFAS, DNA Integrity, and Chromatin

Emerging research continues to shed light on how PFAS affect cellular and molecular systems in ways that extend beyond their persistence and bioaccumulation properties. One of the most concerning aspects of PFAS’s toxicity lies in their capacity to disrupt the structure and function of the genome and epigenome. These disruptions include altered DNA methylation, the induction of DNA damage, alterations in histone modifications, and interference with nuclear receptor signaling, which collectively contribute to genomic instability and chromatin remodeling that can affect how genes are expressed across various biological systems.

### 2.1. PFAS and DNA Methylation

DNA methylation plays a fundamental role in gene expression regulation [15], cellular differentiation [16], and genomic stability [17]. Growing evidence suggests that PFAS exposure can interfere with these methylation processes, leading to broad and sometimes persistent changes in gene regulation.

A whole-genome methylome analysis conducted on MCF-10A human breast epithelial cells revealed that PFOS exposure altered DNA methylation, with over 12,000 differentially methylated CpG sites and more than 2400 affected genes [18]. Notably, tumor suppressor genes, such as *CASC2* and *GACAT3*, became hypermethylated, while oncogenes like *KMT2C* were hypomethylated. In addition, PFOS modified the methylation status of genes involved in estrogen signaling pathways, such as *ESR1* and *GPER1*, suggesting potential disruption of hormone-regulated gene expression [18]. Similar effects have been observed with perfluorooctanoic acid (PFOA), particularly in developing neural cells. In embryonic mouse hypothalamic cells (mHypoE-N46), exposure to PFOA led to a dose-dependent decrease in cell viability and a significant increase in global DNA methylation levels [6]. This hypermethylation was accompanied by the altered expression of key genes involved in apoptosis (e.g., *Bax*, *Casp3*, *Trp73*), cell cycle regulation (e.g., *Ccnb1*, *Ccne1*), and neurotrophic signaling pathways (e.g., *Bdnf*, *Ntrk2*). Moreover, the expression of DNA methylation regulators, including DNA methyltransferases (*Dnmt1*, *Dnmt3a*, *Dnmt3b*) and ten-eleven translocation enzymes (*Tet1* and *Tet3*), was significantly disrupted [6], with marked upregulation of Dnmt1 and Dnmt3a/3b, while Tet1 and Tet3 were downregulated. The expression of the methyl-CpG binding protein 2 (*Mecp2*), a critical reader of DNA methylation marks, was further reduced. In SH-SY5Y neuroblastoma cells, early exposure to low-dose PFOA before differentiation resulted in a persistent reduction in global DNA methylation, suggesting that PFAS can induce stable epigenetic reprogramming in neural cells [19].

Evidence from human studies highlights PFAS-mediated alterations in placental DNA methylation. Prenatal exposure to PFAS has been increasingly associated with epigenetic modifications that may influence early developmental processes, including those related to the nervous system. In a recent epigenome-wide association study, elevated placental concentrations of PFHxS, PFOA, and PFOS were linked to altered DNA methylation at specific CpG sites associated with genes involved in growth, cardiometabolic function, and neurodevelopment, including *NLGN1*, *SHANK2*, and *CNTNAP2* [20]. Although the primary focus of the study was on cardiometabolic outcomes, the involvement of neurodevelopmentally relevant loci suggests that these epigenetic alterations may also have implications for brain development [20]. The placenta is more than a passive barrier; it acts as an endocrine and immune organ that actively integrates maternal environmental exposure and regulates fetal development via hormone production, immune signaling, and epigenetic programming. PFAS accumulation in the placenta can therefore interfere with these regulatory networks, leading to altered DNA methylation profiles, including at loci relevant to neurodevelopment. This supports the Developmental Origins of Health and Disease (DOHaD) hypothesis, which posits that in utero environmental conditions, including toxicant exposures, can shape long-term health trajectories through epigenetic reprogramming. These findings underscore the role of the placenta as a molecular mediator of PFAS-induced effects on the developing brain, highlighting placental epigenetic marks as potential early indicators of neurotoxicity risk [20,21,22,23]. In this context, placental epigenetic changes may serve as early biomarkers of PFAS exposure and fetal neurotoxicity risk, offering valuable tools for early screening, intervention, and the refinement of health-protective thresholds within regulatory frameworks, such as EFSA and REACH [24].

Additionally, PFAS exposure can induce epigenetic reprogramming in sperm by altering DNA methylation patterns, with potential consequences for offspring development. A recent study demonstrated that adult male mice exposed to an environmentally relevant PFAS mixture exhibited changes in sperm DNA methylation, notably at loci implicated in neurodevelopment, including *Ntrk2*, *Bdnf*, and Wnt pathway genes [25]. These alterations were accompanied by persistent transcriptional dysregulation in the liver and adipose tissue of offspring, supporting the hypothesis that PFAS can reprogram developmental pathways via the paternal germline [26]. Although the primary focus was on metabolic outcomes, the involvement of neurogenic loci suggests broader neurodevelopmental implications. These insights also lay the groundwork for a more detailed exploration of PFAS-induced transgenerational effects, which will be addressed in a subsequent section.

### 2.2. PFAS-Induced DNA Damage and Genomic Instability

Beyond their impact on epigenetic regulation, PFAS exposure has also been shown to induce DNA damage, ultimately contributing to genomic instability and impairments in DNA repair mechanisms. This kind of damage is not trivial; it is a key event that can lead to mutagenesis, cancer, and apoptosis [27]. One particularly illustrative example comes from the work of Qin et al. [28], who investigated the effects of perfluorodecanoic acid (PFDA) on DNA integrity and repair mechanisms in ovarian epithelial cells. Their study showed that exposure to low micromolar concentrations of PFDA (1–10 µM), described as environmentally relevant, resulted in the accumulation of DNA double-strand breaks (DSBs) in both primary mouse ovarian epithelial cells and human IOSE-80 cells. While typical human serum levels are around 0.6 nM [24], PFDA’s long half-life and high bioaccumulation potential justify the use of micromolar concentrations to model chronic tissue exposure and detect mechanistic effects, such as DNA damage.

Mechanistically, PFDA was shown to promote the abnormal nuclear accumulation of cyclic GMP-AMP synthase (cGAS), a DNA sensor typically associated with immune signaling [29]. When mislocalized to the nucleus, cGAS interferes with homologous recombination (HR), a key repair pathway, thereby leaving cells more vulnerable to genomic instability. In vivo findings from the same study supported these results, showing increased DSBs and nuclear accumulation of cGAS in the ovarian tissues of PFDA-exposed mice [28]. Although cGAS-mediated interference constitutes one mechanism, oxidative stress emerges as a central driver of PFAS-induced genotoxicity at a broader scale. Several studies have highlighted the potential of PFAS compounds to induce various forms of DNA damage, either directly through oxidative stress (ROS) or indirectly through epigenetic modulation.

For instance, in the study by Mario et al. [4], oxidative-stress-mediated DNA damage was observed in reproductive cells, where exposure to 40 µM in cumulus cells triggered DNA fragmentation and impaired oocyte maturation, pointing to potential reproductive toxicity. Additionally, recent data from *C. elegans* suggest that PFAS exposure may affect mismatch repair (MMR) activity based on altered mutation spectra following chronic exposure [30]. While this points to a potential impact on other DNA repair pathways, further validation in mammalian systems is needed, and current evidence does not demonstrate direct molecular disruption of MMR, base excision repair (BER), or non-homologous end joining (NHEJ) components.

These effects extend to hepatic models, where PFAS mixtures have been shown to induce genotoxicity and epigenetic dysregulation in human liver cells (HepG2), reinforcing concerns regarding the cumulative toxicological effects of multiple PFAS exposures [31]. Although short-chain PFAS exposure, once considered less harmful, has been found to significantly increase oxidative stress biomarkers across several human cell lines without inducing direct DNA strand breaks; this suggests a more upstream mechanism for genomic instability [32]. Similarly, Pierozan et al. [33] reported that low concentrations of PFOS and PFOA mixtures (100–500 pM) synergistically induced not only oxidative stress and DNA/RNA damage but mitochondrial dysfunction in human breast epithelial cells, further supporting the idea that PFAS mixtures may present a cumulative genotoxic risk. In reproductive cells, PFOS (600 µM) and PFOA (400 µM) exposure during mouse oocyte maturation led to an increase in γ-H2AX levels, a marker of DNA double-strand breaks, with damage severity correlating with carbon chain length and the presence of a sulfonate group [34].

Although most of these studies center on hepatic, epithelial, and reproductive systems, emerging evidence indicates that the nervous system is not spared. In differentiated SH-SY5Y neuronal cells, PFAS mixtures led to increased production of ROS and mitochondrial dysfunction at concentrations ranging from 10 to 100 µM, both key early events in oxidative stress responses [35]. Comparable findings in microglial cell lines confirmed that even short-chain PFAS, such as PFBS, triggered oxidative stress at concentrations of 10–50 µM [32]. Additional work in SH-SY5Y cells showed disrupted redox and metabolic profiles after exposure to PFOA and PFBA (10–100 µM) [36,37], reinforcing the idea that PFAS can compromise neuronal oxidative homeostasis. However, it is important to note that while these studies consistently show oxidative imbalance, direct measurements of DNA damage, such as strand breaks or chromatin instability, were not performed. One notable exception is the study by Running et al. [5], in which neurons exposed to 10 µM PFOS and PFOA exhibited transcriptomic and lipidomic perturbations indicative of oxidative stress and altered lipid metabolism, two hallmarks often linked to genomic instability. However, no direct evidence of DNA damage was demonstrated. These findings underscore a significant knowledge gap. Although PFAS clearly induce DNA damage in neuronal cells, the extent to which this occurs remains unclear. To address this, future studies should incorporate direct genotoxicity assays, such as the comet assay or γ-H2AX staining, in neural models. This will be essential to confirm whether oxidative stress induced by PFAS exposure translates into measurable DNA damage in neural cells, ultimately contributing to neurotoxicity.

Although standard genotoxicity assays, such as the comet assay, γ-H2AX staining, and the micronucleus test, are widely used, they face important limitations when applied to PFAS research. One key issue is that many PFAS compounds induce oxidative stress, which may lead to false positives in DNA damage assays [38]. For instance, the comet assay may detect strand breaks that are the result of ROS rather than direct genotoxicity [39]. Another common problem in vitro is the limited bioavailability of PFAS, as they tend to bind to proteins in the culture medium or adsorb to plastic surfaces, potentially masking intracellular effects [40]. These challenges are especially relevant in neuronal systems. Most mature neurons are post-mitotic and therefore unsuitable for micronucleus assays, which rely on cell division [41]. In addition, γ-H2AX staining in neurons can result in pan-nuclear patterns associated with apoptosis or general chromatin stress, rather than bona fide DNA double-strand breaks [42]. That said, when using proliferative neural models, such as neural stem cells or progenitors, micronucleus assays and cell-cycle-dependent markers remain relevant and can provide valuable genotoxicity information.

To improve assay specificity and sensitivity in neuronal contexts, future studies should prioritize discrete γ-H2AX foci counting and integrate measuring oxidative DNA lesions like 8-hydroxy-2′-deoxyguanosine (8-OHdG). Further refinements include using serum-free media to enhance PFAS uptake, applying chronic low-dose exposures that reflect environmentally relevant concentrations, and pairing genotoxic endpoints with transcriptomic profiling of DNA repair and stress response pathways. These adaptations will enhance the mechanistic resolution and translational relevance of PFAS genotoxicity testing in neural systems.

### 2.3. PFAS and Chromatin Structure Modifications

Beyond their ability to induce DNA methylation changes and promote oxidative DNA damage, PFAS have also been implicated in the modulation of chromatin architecture through alterations in histone post-translational modifications. These modifications play a pivotal role in determining chromatin accessibility and transcriptional activity, thereby influencing gene expression at a fundamental level. In human breast epithelial cells, Pierozan et al. [43] showed that exposure to PFOS and PFOA significantly disrupted histone landscapes. Their Western blot analyses revealed a reduction in H3K9 acetylation—a mark typically associated with active chromatin—alongside alterations in H3K9 demethylation and H3K4 trimethylation, both of which regulate chromatin accessibility and gene silencing. Importantly, these changes were accompanied by transcriptional dysregulation of key cell cycle regulators. PFOS exposure increased levels of *cyclin D1 (CCND1)* and *CDK4* while reducing the expression of the cell cycle inhibitors *p21 (CDKN1A)*, *p27 (CDKN1B)*, and *p53*, which promoted G_0_/G_1_-to-S phase progression. In neural systems, similar patterns of disruption have been observed. DA-like neurons derived from SH-SY5Y neuroblastoma cells and exposed to PFOA exhibited a persistent depletion of bivalent histone marks H3K4me3 and H3K27me3 detected via immunofluorescence and chromatin profiling [19]. These marks are known to maintain a poised transcriptional state, and their depletion suggests potential gene expression reprogramming in neuronal contexts.

Critically, this line of evidence extends from in vivo models to human populations. Tsai et al. reported that prenatal exposure to PFAS—including PFOA, PFNA, and PFUA—was associated with altered global histone methylation profiles in two-year-old children [44]. Specifically, increased levels of the active mark H3K4me3 and decreased levels of the repressive mark H3K27me3 were observed in peripheral blood leukocytes, suggesting a shift toward transcriptionally active chromatin states early in life [44].

Adding another layer of concern, recent research comparing the effects of PFOA and its replacement compound HFPO-TA revealed that both substances enhanced the expression of key steroidogenic genes (*Star* and *Cyp11a1*) in MLTC-1 cells via chromatin modulation. This occurred through changes in H3K4 and H3K9 methylation, with HFPO-TA exerting even stronger effects than PFOA, highlighting that this alternative compound may pose equal or greater risks to male reproductive health through similar epigenetic mechanisms [45]. Together, these studies point to histone dysregulation as a conserved epigenetic mechanism through which PFAS may reprogram gene expression across diverse biological systems and developmental windows. Further studies are warranted to elucidate how these changes in histone marks intersect with broader chromatin dynamics, particularly in the developing brain.

### 2.4. PFAS and Nuclear-Receptor-Mediated Chromatin Disruption

PFAS are increasingly recognized for their ability to modulate nuclear receptor (NR) signaling, a vital process that regulates metabolism, hormone balance, and xenobiotic regulation. In liver-derived models, compounds like PFOA have been shown to activate key nuclear receptors like PPARα, PPARγ, and estrogen receptors, leading to transcriptomic shifts consistent with nuclear-receptor-mediated toxicity [7]. Notably, the strength and specificity of this activation depend heavily on the chemical structure of PFAS molecules. Factors like the length of the fluorocarbon chain and the nature of the head group significantly influence receptor binding and potency [46,47]. Computational screening has further expanded this understanding, predicting that thousands of PFAS and their mixtures may bind to orthosteric or allosteric sites across multiple nuclear receptor families—including PPARs, CAR, FXR, and ERα—suggesting a complex, structure-driven toxicological profile [48]. Of particular concern is PPARγ, which appears highly sensitive to PFAS interference. Detailed structural analysis has revealed that PFAS can bind to the ligand-binding domain of PPARγ, thereby altering chromatin regulation and downstream gene transcription [49]. Moreover, a secondary, noncanonical binding site on PPARγ was first identified through computational modeling, suggesting that PFAS may amplify transcriptional disruption via allosteric modulation [50]. This mechanistic hypothesis was substantiated by structural studies showing that PFOA engages multiple ligand-binding pockets, including one near the activation function-2 (AF-2) surface critical for coactivator recruitment, thereby supporting its partial agonist activity [8]. These computational predictions are further supported by high-throughput screening data from the ToxCast program, which demonstrate that common PFAS, such as PFOA, PFOS, PFNA, and PFHxS, activate PPARα and PPARγ with AC_50_ values typically in the 1–10 µM range [51].

These findings highlight nuclear receptor interference as a conserved and central mode of action of PFAS, with implications that likely extend beyond hepatic systems to other receptor-rich tissues, including the brain.

Critically, emerging evidence suggests that PFAS-induced activation of nuclear receptors, such as PPARγ, may trigger epigenetic remodeling. Beyond simply regulating transcription, PPARγ plays an active role in modifying the chromatin environment [52] by recruiting chromatin-modifying enzymes, such as CBP/p300 and HDACs, which govern key post-translational histone modifications [53]. These enzymes dynamically reshape chromatin’s structure by adding or removing chemical tags, thereby influencing transcriptional accessibility [54]. This positions PPARγ as a critical link between environmental signals and epigenetic reprogramming. Given its established role in neuronal differentiation and plasticity, PFAS-mediated disruption of PPARγ signaling may have profound consequences for neurodevelopment and brain function [55]. Supporting this hypothesis, Wan Ibrahim et al. demonstrated that PFOS exposure altered PPARγ expression both in vitro in rat neural stem cells and in vivo in fetal brain tissue, resulting in abnormal differentiation patterns and reduced neural stem cell proliferation [56]. More broadly, nuclear receptors appear to serve as molecular bridges between environmental exposure and chromatin-level changes, making them key regulators in the epigenetic response to PFAS. This is particularly relevant in the context of neurotoxicity, where further investigations are needed to validate these mechanisms within neuronal systems and understand their contribution to long-term brain dysfunction.

## 3. Role of Epigenetics and Genotoxicity in PFAS-Induced Neurotoxicity

Developmental exposure to PFAS has been linked to long-lasting neurotoxic effects, including impaired neuronal differentiation, disrupted synaptic plasticity, and cognitive deficits [1,2,57]. Studies from both in vitro neural models and in vivo animal studies point to early-life PFAS exposure as a trigger for persistent epigenetic modifications [58,59], raising critical concerns about the potential for transgenerational transmission of neurotoxic outcomes [58,60]. Notably, these enduring effects cannot be fully attributed to PFAS bioaccumulation alone in the body, as blood levels decline over time while molecular and behavioral changes persist [3,61]. Furthermore, emerging data show that PFAS exposure can alter synaptic signaling, neurotrophic factor regulation, and inflammatory pathways in differentiating neurons, even after toxicant removal [1,2,3,57]. For instance, a study by Running et al. [5] demonstrated that PFOA exposure significantly altered the expression of nearly 600 genes in SH-SY5Y neuronal-like cells, including those involved in synaptic growth and neural function. Of particular concern, *MANF* (mesencephalic astrocyte-derived neurotrophic factor), crucial for neuronal survival, was consistently downregulated, while *TXNIP* (thioredoxin-interacting protein), which is associated with neuronal apoptosis, was upregulated. These changes persisted beyond the exposure period, indicating long-term effects on neuronal gene expression.Given the growing body of evidence linking PFAS exposure to epigenetic dysregulation and genomic instability, understanding how these upstream molecular impairments contribute to neurodevelopmental disorders, cognitive disruption, and neurodegenerative diseases is pivotal for assessing long-term risk. This section explores how such early disturbances in DNA methylation, histone modifications, and genome integrity may reprogram neural gene expression and leave a lasting imprint on brain development. These upstream perturbations are increasingly implicated in the disruption of key neurodevelopmental endpoints, including BDNF signaling, synaptogenesis, neuroinflammation, mitochondrial function, calcium homeostasis, and neurotransmitter regulation, all of which are critical to maintaining cognitive and neurological function.

### 3.1. BDNF as a Downstream Target of Epigenetic and Genotoxic Disruption

BDNF plays a central role in regulating neuronal differentiation, synaptic plasticity, and cognitive function [62]. Its expression is finely controlled by epigenetic mechanisms, including DNA methylation, histone modifications, and microRNA (miRNA)-mediated regulation [63,64,65,66]. Disruptions in BDNF signaling have been reported following exposure to several environmental neurotoxicants. For instance, in rodent models, repeated low-dose exposure to the organophosphate diisopropylfluorophosphate (DFP) altered hippocampal histone acetylation and led to Bdnf downregulation [67]. Similarly, long-lasting reductions in Bdnf expression were observed in brain tissue after perinatal exposure to methylmercury [68]. In juvenile mice, co-exposure to manganese and lead induced synergistic repression of hippocampal Bdnf expression via histone modifications and DNA methylation [69]. These alterations have been associated with both neurodevelopmental and neurodegenerative disorders [67,70], positioning BDNF as a particularly sensitive and informative target for evaluating the neurotoxic potential of PFAS.

Recent in vitro evidence supports the idea that PFAS, especially PFOS, may suppress BDNF expression through epigenetic mechanisms [71,72]. In human neuroblastoma SK-N-SH cells, PFOS exposure has been shown to significantly reduce BDNF mRNA and protein levels, coinciding with increased methylation at BDNF promoters I and IV [71]. This hypermethylation was accompanied by shifts in DNA methyltransferase activity; PFOS decreased DNMT1, a maintenance methyltransferase, while increasing DNMT3B, responsible for de novo methylation, suggesting a repressive epigenetic shift in BDNF transcription [71]. In parallel, PFOS also altered microRNA profiles, particularly upregulating miR-16, miR-22, and miR-30a-5p, all of which target BDNF transcripts, thereby reinforcing gene silencing through post-transcriptional repression [71]. Functionally, these epigenetic disruptions impair BDNF–ERK–CREB signaling. In SH-SY5Y cells, PFOS exposure leads to a reduction in both phosphorylated ERK (p-ERK) and BDNF levels while paradoxically increasing phosphorylated CREB (p-CREB) [72]. These signaling imbalances are further accompanied by dose-dependent modulation of TrkB BDNF’s high-affinity receptor, which is upregulated at lower PFOS concentrations but suppressed at higher doses [72]. Among the implicated regulators, miR-22 appears particularly important, as its upregulation has been directly linked to decreased BDNF expression and impaired ERK-CREB signaling. Together, these data suggest that PFOS-mediated epigenetic regulation disrupts both the transcription and functional signaling of BDNF, potentially contributing to long-lasting neurodevelopmental deficits.

Beyond direct epigenetic regulation, NRF2 (Nuclear factor erythroid 2-related factor 2) dysfunction emerges as a key mediator of PFAS-induced BDNF suppression. As a transcription factor critical for neuronal homeostasis, synaptic plasticity, and neurotrophic signaling [73,74], NRF2 promotes BDNF transcription by repressing epigenetic silencing factors, such as HDAC2, mSin3A, and MeCP2, thereby facilitating chromatin accessibility at BDNF promoter regions and thus enhancing neuroprotection [75]. This pathway appears particularly vulnerable to oxidative stress induced by PFAS. In both mouse astrocytes and zebrafish embryos, PFOS exposure has been shown to inhibit Nrf2 activation and increase ROS, implicating the Nrf2–MAPK axis in PFAS-mediated toxicity [76,77]. Given NRF2’s dual role in maintaining BDNF expression and regulating neuroinflammatory tone, its suppression by PFAS may contribute to impaired neurodevelopment by simultaneously reducing neurotrophic support and exacerbating oxidative damage. Evidence from neurodegenerative models reinforces this link. In Parkinson’s disease, for instance, epigenetic silencing of *NRF2*, e.g., via lncRNA MALAT1, has been shown to enhance inflammasome activation and progressive neuronal loss, further illustrating the sensitivity of NRF2-BDNF signaling to chromatin-level dysregulation [78]. Similarly, in the Alzheimer’s disease model, DNA demethylation of the *NRF2* promoter restores *NRF2* expression and neuroprotection [79]. Taken together, these findings illustrate how PFAS can disrupt BDNF signaling through both epigenetics and oxidative pathways, with *NRF2* suppression emerging as a key mediator. However, the mechanistic complexity of PFAS toxicity suggests that additional layers of regulation may be involved. In particular, the potential contribution of genotoxic mechanisms to BDNF dysregulation remains underexplored. Although no studies to date have directly linked PFAS-induced DNA damage to alterations in BDNF signaling, both pathways, epigenetic disruption and genomic instability, have independently been implicated in PFAS-related neurotoxicity. Given BDNF’s sensitivity to genomic integrity and its regulation by chromatin’s structure, it is plausible that genotoxic stress may converge with epigenetic alterations to influence its expression. This convergence could further exacerbate neurodevelopmental vulnerability, particularly during early-life windows of heightened plasticity. Future studies should explore this interaction explicitly.

### 3.2. PFAS-Induced Interference with Synaptic and Neurotransmitter Gene Networks

Emerging data suggest that synaptic development is particularly vulnerable to environmental disruption at the epigenetic level, including that caused by PFAS exposure. In zebrafish, PFNA was shown to impair gene networks critical to synaptogenesis, leading to deficits in neuronal connectivity and cognitive function [80]. Transcriptomic profiling has identified several key synaptic genes as targets of PFNA neurotoxicity. Notably, DLG4 (PSD-95), a postsynaptic scaffolding protein critical for excitatory synaptic structure, was significantly downregulated, suggesting impaired postsynaptic assembly. Likewise, *Grin2b*, which encodes the NR2B b subunit of the NMDA receptor and regulates calcium-dependent synaptic plasticity, was suppressed, potentially compromising activity-driven synapse maturation. On the presynaptic side, PFNA reduces the expression of *Syn1* (Synapsin I), a key regulator of vesicle mobilization and neurotransmitter release [80].

While these transcriptional changes are well-documented, direct evidence linking PFAS exposure to epigenetic or genotoxic alteration at synaptic gene loci remains limited. Nonetheless, the regulation of synaptic genes is known to be highly responsive to histone acetylation and DNA methylation [81,82], both of which control activity-dependent gene expression essential for synaptic remodeling [83,84]. This raises a strong mechanistic possibility that PFAS-induced chromatin changes may contribute to impaired synaptic plasticity. Supporting this, persistent DNA damage in a Huntington’s disease model has been shown to suppress the expression of core synaptic plasticity genes, such as *Bdnf*, *Arc*, and *Egr1*, linking genomic instability to impaired synaptic function [85]. Given PFAS-associated oxidative and genotoxic stress [25,28,31], these findings support a mechanistic hypothesis wherein PFAS may indirectly suppress synaptic gene expression via both epigenetic and DNA-damage-associated pathways. Future studies should directly assess chromatin regulation at synaptic loci following PFAS exposure to better define its neurodevelopmental consequences.

In parallel with its effects on synaptic architecture, PFAS may also disrupt neurotransmitter systems, further compromising neuronal communication through epigenetically mediated pathways. PFOS and PFOA, in particular, have been implicated in the disruption of neurotransmission, with a notable impact on the dopaminergic and glutamatergic systems. Experimental studies report that developmental exposure to these compounds reduces dopamine levels in zebrafish and rodents while elevating hippocampal glutamate in rats, potentially triggering excitotoxicity and increasing vulnerability in dopaminergic neurons [59,86]. In developmental models, frog larvae exposed to 10–1000 ppb PFAS showed a clear dose–response reduction in dopamine levels and increased turnover, highlighting a critical window of vulnerability during early neurodevelopment [87]. Beyond these effects, PFAS have also been shown to interfere with GABAergic signaling. For example, PFOS inhibits human GABAA receptors with an IC_50_ of approximately 28 µM, producing long-lasting effects, while PFOA exhibits similar but rapidly reversible inhibition at an IC_50_ of ~6.7 µM [88].

While these findings position neurotransmitter system disruption as a core outcome of PFAS exposure, the underlying regulatory mechanisms remain incompletely understood. Notably, epigenetic processes have emerged as key modulators of neurotransmission in other neurotoxic or neuropsychiatric contexts, providing a mechanistic framework that may also apply to PFAS. For instance, NMDA receptor hypofunction in schizophrenia has been linked to DNA methylation and histone modifications at glutamate receptor subunit genes in humans [89], and methamphetamine exposure reduces striatal glutamate receptor expression via histone deacetylation and miRNA dysregulation in rodents [90]. Similarly, the differentiation of dopaminergic neurons is epigenetically regulated, with transcription factors, such as *Nurr1* and *Pitx3*, subject to methylation-sensitive control, as reported in human and rodent studies [91].

Findings from other environmental toxicants further reinforce this model. Methylmercury, a well-characterized developmental neurotoxicant, has been shown to disrupt neurotransmitter-related gene expression through promoter methylation and chromatin alterations [68], whereas PBDE-99 impairs cholinergic signaling by altering the transcription of neurotransmitter biosynthesis genes during early brain development [92]. Similarly, atrazine interferes with dopaminergic signaling through miRNA-mediated regulation and AMPK-dependent epigenetic pathways [93]. Despite sharing epigenetic modes of action, these toxicants differ mechanistically, with PFAS exhibiting a notably broader profile, particularly through their direct modulation of nuclear receptors, such as PPARγ. For example, hydroxylated PBDE metabolites like 3-OH-BDE-47 can bind PPARγ [94], but PFAS, such as PFOA and PFOS, show both orthosteric and allosteric binding to PPARγ, leading to chromatin remodeling via recruitment of co-regulators, such as CBP/p300 and HDACs. In contrast, methylmercury modulates PPAR γ expression indirectly, likely via oxidative stress and promoter methylation [95], while atrazine shows no affinity for PPAR isoforms and instead acts through endocrine and miRNA-mediated pathways [96]. These distinctions highlight that although all of these toxicants impact neurotransmission and neurodevelopment, PFAS uniquely couple receptor-level activation with chromatin remodeling, thereby contributing to persistent transcriptional dysregulation.

Taken together, these observations suggest that PFAS may impair neurotransmitter homeostasis not only through receptor or metabolic interference but also through epigenetic mechanisms. Dedicated studies are needed to map the chromatin landscape and transcriptional regulation of neurotransmission under PFAS exposure, particularly during critical windows of neurodevelopment.

### 3.3. Neuroinflammation and Immunoepigenetic Disruption by PFAS

Growing evidence positions PFAS, particularly PFOS, as potent modulators of neuroinflammatory responses. These effects appear to result from a complex interplay between immune activation, oxidative stress, and epigenetic regulation. At the molecular level, PFOS exposure has been shown to activate glial-mediated neuroinflammation, notably through the upregulation of pro-inflammatory cytokines, such as TNF-α, via the JAK2/STAT3 signaling pathway [97]. Supporting this, comparative proteomic analyses reveal that even newer-generation PFAS like GenX trigger astrocyte inflammation and metabolic dysfunction, marked by the altered expression of genes involved in mitochondrial respiration, cytokine production, and immune signaling—including IL-6 upregulation and reduced expression of redox-regulatory genes—suggesting a broader class-wide effect on glial reactivity [98]. In vivo studies in larval zebrafish reveal that PFOS exposure sensitizes microglia to injury, increasing the expression of the microglial marker *p2ry12* and enhancing immune vigilance in the brain [99]. Astrocyte-specific responses are similarly impacted, with PFOS and PFOA impairing NRF2-mediated antioxidant signaling and thereby exacerbating oxidative stress, a known trigger for inflammatory activation in the brain [100].

Beyond the CNS, systemic inflammation adds another layer of concern. Subchronic PFOS exposure in adult mice increases systemic levels of IL-6 and TNF-α, while additional studies show that PFAS pollutants engage the innate immune system through AIM2 inflammasome activation, pointing to inflammasome priming as a potential mechanistic bridge between PFAS exposure and neuroinflammation [101]. In parallel, the NF-κB/TNF-α pathway, widely implicated in inflammation-related pathology, has been shown to be activated by PFOS in both hepatic and neural tissues, highlighting a conserved pro-inflammatory cascade across organ systems [97,102]. Notably, when PFOS exposure occurs prenatally and is combined with a high-fat diet, genomic reprogramming of neuromotor pathways has been observed in offspring, suggesting that early-life exposure may leave lasting susceptibility to neuroinflammation and behavioral dysfunction [103].

Neuroimmune activation is increasingly understood through the lens of epigenetic regulation. Human studies demonstrate that PFAS exposure is associated with differential DNA methylation at key immunoregulatory loci, including *RUNX3* (a transcription factor involved in T-cell differentiation and cytokine signaling), *NFKBIA* (which encodes IκBα, a negative regulator of the NF-κB pathway), and *IL6R* (the interleukin-6 receptor mediating inflammatory signaling), as well as signatures of accelerated epigenetic aging in occupationally exposed individuals [104]. In utero PFOS exposure in mice has been shown to induce epigenomic remodeling in fetal liver, with differential expression of genes, such as *Ccl2* (encoding the chemokine MCP-1, involved in recruiting monocytes to inflammation sites), *Il1rn* (interleukin-1 receptor antagonist, a regulator of IL-1 signaling and inflammation), and *Tnfrsf12a* (also known as Fn14, a receptor for TWEAK, involved in TNF superfamily signaling and glial inflammation), all of which play critical roles in cytokine signaling and glia inflammation [105]. These findings reinforce the plausibility of developmental programming via PFAS-induced chromatin changes.

While direct causal links between PFAS-induced epigenetic alteration and neuroinflammation remain to be fully established, there is strong mechanistic support from the adjacent literature. Histone modifications, such as H3K27ac, have been implicated in microglial activation and transcriptional reprogramming [106], while dynamic DNA methylation is known to regulate inflammatory gene expression in both astrocytes and microglia [107,108]. Epigenetic modulation of autophagy and cytokine signaling genes in glia has been shown to influence neuroinflammatory tone, suggesting that PFAS-induced chromatin remodeling may converge on similar regulatory pathways [109].

These insights reveal chromatin remodeling as a key mechanistic bridge between PFAS exposure and neuroimmune dysregulation, with implications that span both development and long-term neurotoxicity.

## 4. PFAS, ncRNA Dysregulation, and Neurotoxicity

ncRNAs, including miRNA and long non-coding RNAs (lncRNAs), are emerging as key mediators of PFAS-induced neurotoxicity. These molecules are central to neural development and function, and mounting evidence indicates that PFAS exposure alters their expression and regulation [110].

Epidemiological studies, such as those conducted in firefighters and adolescents, have identified PFAS-associated changes in plasma miRNA profiles, implicating key extracellular miRNAs in inflammatory and metabolic-related pathways [9,111,112]. These findings highlight not only the systemic reach of PFAS but also the potential utility of circulating miRNA as a minimally invasive biomarker of exposure and effect. Parallel data from a pilot study in children and in utero exposure models further support the role of PFAS in modulating miRNA expression during critical developmental windows, raising legitimate concerns about long-term effects on fetal growth and neurodevelopment [113,114].

Mechanistic studies have shown that PFOS disrupts miRNA profiles in human iPSC-derived neurons and PC12 cells, with consistent disruption of neurotrophic and apoptotic signaling pathways. In iPSC-derived neurons, PFOS exposure alters the extracellular miRNA profile in a manner consistent with early neurodegenerative changes [10]. Although specific miRNAs were not fully characterized in this system, complementary studies in PC12 cells have revealed that PFOS activates the pro-apoptotic transcription factor FoxO3a, leading to increased expression of Bcl-2 family proteins, such as Bim and Bax [115]. Concomitantly, *miR-22* was significantly upregulated and directly targets BDNF transcripts for repression, even when CREB is phosphorylated, suggesting a miRNA-mediated block in BDNF signaling [71]. Meanwhile, *miR-16*, also involved in BDNF regulation and cell cycle control, is downregulated, potentially reflecting a failed compensatory mechanism that exacerbates cellular vulnerability [71].

Animal studies provide further mechanistic insight into PFOS toxicity. Gestational PFOS exposure alters miRNA and lncRNA profiles in fetal rat brains and human trophoblasts, impairing synaptic development and angiogenesis [116,117,118]. Notably, *MEG3*, *XIST*, and *H19*, three epigenetically regulated lncRNAs, have been implicated in placental growth inhibition and neurodevelopmental dysregulation. These lncRNAs act either as scaffolds for chromatin modifiers or as miRNA sponges, indirectly modulating gene networks involved in cellular survival and differentiation. For instance, the *MEG3/miR-770/PTX3* axis and the *H19/miR-19a/19b* module have been shown to mediate PFOS toxicity by targeting inflammatory and growth-related genes [116,119].

PFOS induces epigenetic silencing of *H19* via promoter hypermethylation, leading to reduced expression of its associated miRNAs *miR-19a* and *miR-19b*, which are crucial regulators of apoptosis and angiogenesis [116]. The loss of *H19* not only reduces their direct gene-regulatory effects but also disrupts their role as competing endogenous RNA (ceRNAs), further amplifying dysregulation in target mRNA pathways. While predominantly studied in placental tissues, the *H19/miR–19a/b/PTEN* axis is also active in the developing brain [120,121,122,123], suggesting a conserved mechanism underlying PFOS-induced neurotoxicity.

Similarly, *MEG3* is epigenetically repressed by PFOS via promoter hypermethylation, as demonstrated in placental models [119], thereby downregulating its intron product, *miR-770*, which normally suppresses *PTX3*, an inflammatory mediator. The resulting overexpression of *PTX3* contributes to both growth inhibition and inflammatory stress, effects mirrored in neural tissues, where both *MEG3* and *PTX3* are expressed in neural tissues, and similar regulatory roles for *MEG3* in neuronal apoptosis and glial inflammation have been described [119,124,125,126]. This finding suggests that the *MEG3/miR-770/PTX3* axis may also be relevant to PFAS-induced neurotoxicity through inflammatory and epigenetic pathways.

System-level analysis adds to this picture. Integrated miRNA transcriptomics and proteomics have provided a broader perspective on ncRNA dysregulation due to PFAS exposure. In a mouse model of PFNA exposure, coordinated repression of miRNAs alongside hepatotoxic proteins upregulation was observed, revealing ncRNA involvement in both direct organ toxicity and systemic metabolic effects [127]. Notably, several of these altered genes and miRNAs, such as those regulating oxidative stress (*SOD1*, *GPx*) [128], inflammation (*miR-155*, *miR-21*) [11,129], and metabolic control (*PPARα*) [130], are also expressed in neural tissues. This reinforces the notion of interconnected regulatory mechanisms where PFAS-induced ncRNA dysregulation may exert systemic effects, with overlapping mechanisms in the liver and brain, particularly via pathways controlling redox balance, glial inflammation, and apoptosis. To our knowledge, however, integrated miRNA–proteomic analyses remain primarily focused on hepatic tissues, with no systematic studies conducted to date in PFAS-exposed brain tissue or neuronal models.

Based on the evidence presented above, a coherent mechanistic model emerges. PFAS-induced neurotoxicity stems from epigenetic disruption of ncRNA networks essential for neurodevelopmental homeostasis. Hypermethylation of key lncRNAs (*H19*, *MEG3*) impairs miRNA-dependent regulation of inflammatory and apoptotic pathways, involving critical targets, such as *PTEN* and *PTX3*. Concurrently, disruption of the *miR-22/miR–16 axis* affects BDNF translation. This cascade impairs neurotrophic signaling, angiogenesis, and immune balance, with systemic implications, positioning ncRNA dysregulation as a central and actionable mechanism in PFAS neurotoxicity.

## 5. Transgenerational Effect of PFAS Exposure

Although direct evidence linking PFAS-induced transgenerational epigenetic changes to brain-specific outcomes remains limited, multiple experimental models have demonstrated that early-life PFAS exposure can reprogram the germline, resulting in heritable alterations in gene expression across generations. While these effects are most consistently observed in metabolic and reproductive tissues, several studies suggest that neurodevelopmental pathways may be indirectly impacted. This section explores current insights into PFAS-induced epigenetic inheritance and its potential relevance to brain development across generations.

Animal studies have revealed that exposure to PFAS during critical developmental windows can induce transgenerational effects by altering DNA methylation in germ cells. For example, in a mouse model, maternal exposure to a PFAS mixture (PFOA, PFOS, PFHxS, PFNA) at 2.5 mg/L each in drinking water—a concentration relevant to environmental exposure—resulted in persistent DNA methylation changes in F0 sperm. These epimutations were associated with long-term transcriptomic reprogramming in the liver and adipose tissue of F1 and F2 offspring [25]. Key differentially methylated regions mapped genes involved in lipid metabolism (Pparα and Acsl1), immunity, and developmental patterning (Hox clusters) [14]. Notably, these effects were transmitted across generations in the absence of continued PFAS exposure, emphasizing the role of stable epigenetic inheritance.

In human in vitro models, PFOS exposure (1–10 µM) in spermatogonial stem cells led to the downregulation of key spermatogenic genes, including *SYCP3*, *PRM1*, and *DAZL*, and altered chromatin markers, including a loss of H3K4me3 and H3K9me2 (a heterochromatin-associated mark) and a reduction in global 5-methylcytosine levels [131]. These chromatin changes suggest impaired germ cell integrity and potential disruption in the transfer of epigenetic information [26]. Interestingly, supplementation with S-adenosylmethionine (SAM), a universal methyl donor, has been shown to partially restore DNA methylation balance and ameliorate PFOS-induced toxicity, highlighting possible avenues for intervention [132].

Translational models like zebrafish and *C. elegans* support these observations and offer mechanistic insights into heritable toxicity. In *C. elegans*, PFAS disrupted nuage structures and RNA granules, such as P granules, critical for RNA silencing and small RNAs’ regulation during spermatogenesis, with downstream consequences for piRNAs’ and miRNAs’ localization and germline inheritance [133]. Furthermore, low-dose exposure to PFBS and PFHxS (0.1–10 µM) altered the expression of *fat-7* and *ech-1*, key regulators of fatty acid metabolism, along with changes in the localization and activity of the NHR-49 nuclear hormone receptor, suggesting persistent metabolic reprogramming [12,134]. Additional work in this model has demonstrated germline DNA damage, reproductive defects, and behavioral anomalies persisting into the F2 generation, including alterations in locomotion and chemotaxis, pointing to disrupted neuronal reprogramming [128,129].

In zebrafish, developmental PFAS exposure led to gene expression changes in inflammation, neurodevelopment, and oxidative stress pathways in F1 and F2 offspring [58], These heritable gene expression shifts were observed in pathways like cytokine signaling and mitochondrial function, raising the possibility that early-life PFAS exposure can epigenetically program long-term neuroimmune sensitivity across generations. Although the precise epigenetic carriers, such as DNA methylation marks or histone modifications, were not directly identified, the persistence of transcriptomic changes in the absence of continued exposure strongly implicates heritable chromatin alterations or disrupted germline epigenetic reprogramming as key drivers.

Complementing these findings, Cui et al. demonstrated that chronic low-level PFOS exposure in adult zebrafish (F0) led to hepatic steatosis and structural abnormalities in liver and intestinal tissues. In their offspring, genes like *lepa*, *kiss1*, *dgat1b*, and *hb9* showed altered expression, with implications for neuroendocrine regulation [135]. Similar findings were reported with F-53B, a PFOS analog, where parental exposure induced hepatotoxicity and—critically—altered the PPAR signaling pathway in unexposed offspring [136]. Given the central role of PPARs in lipid metabolism and inflammatory gene regulation, these findings suggest that PFAS analogs may epigenetically rewire key metabolic-inflammatory circuits across generations.

PFAS-induced epigenetic effects also appear to extend to endocrine and reproductive systems. In fish, developmental exposure to PFBS has been shown to skew sex ratios, suggesting interference with early gonadal programming and sex hormone pathways [137]. Combined parental exposure to PFOS and ZnO nanoparticles resulted in stress-related gene expression shifts in offspring, underscoring cumulative effects on endocrine and developmental signaling [13]. Disruption of thyroid hormone homeostasis by PFAS—widely reported in recent reviews—is especially concerning, as thyroid hormones play a critical role in brain development and metabolism [134,135,138].

Although some of these studies did not directly assess specific epigenetic marks, the persistence of transcriptional dysregulation and phenotypic changes strongly supports the presence of heritable regulatory alterations. Zebrafish, in particular, have proven to be a powerful model for detecting compound-induced changes in DNA methylation during embryogenesis [139]. In one such study, Kim et al. demonstrated that developmental exposure to an understudied PFAS analog, 8:8 perfluoroalkyl phosphinic acid, led to significant global DNA methylation changes accompanied by neurobehavioral impairments, including, notably, reduced locomotor activity, establishing a direct link between epigenetic disruption and neurodevelopmental phenotype [140].

Altogether, PFAS-induced transcriptomic changes in offspring, such as those affecting PPAR pathways, are likely driven by disrupted DNA methylation dynamics established during the developmental period and maintained across generations. These findings position epigenetic disruption as a core mechanism through which PFAS may exert long-term, transgenerational effects. While definitive proof of brain-specific, heritable toxicity is still evolving, converging data from transcriptomic, behavioral, and germline studies suggest plausible links between PFAS exposure and lasting changes in neurodevelopmental trajectories. Further studies incorporating a multigenerational profile of neural tissues are needed to clarify these mechanisms and assess potential risks.

**Figure 1:**
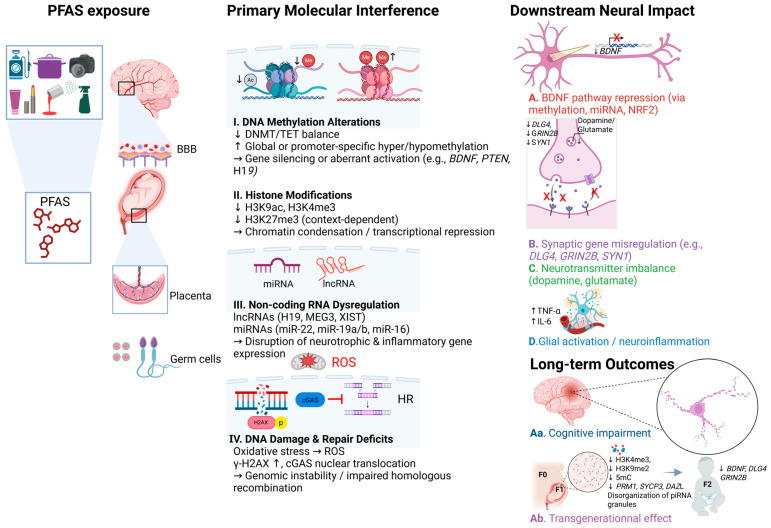
Mechanistic overview of PFAS-induced neurotoxicity and potential transgenerational effects. PFAS compounds, derived from various environmental and consumer sources, cross biological barriers such as the placenta and the blood–brain barrier (BBB), reaching sensitive tissues, including the brain and germ cells. At the molecular level, PFAS exposure disrupts epigenetic and genomic regulation through several key mechanisms: (**I**) DNA methylation alterations, including hypermethylation or hypomethylation of neurodevelopmental genes (e.g., *BDNF*, *PTEN*, *H19*) via DNMT/TET imbalance; (**II**) histone modification changes, such as altered H3K4me3, H3K9ac, and H3K27me3 levels, affecting chromatin accessibility; (**III**) dysregulation of non-coding RNAs (miR-22, miR-19a/b, miR-16; lncRNAs *H19*, *MEG3*, *XIST*), impacting neurotrophic and inflammatory gene expression; and (**IV**) DNA damage and repair deficits, including γ-H2AX phosphorylation and cGAS nuclear translocation, leading to genomic instability and impaired homologous recombination. These upstream events result in downstream neural consequences: (**A**) repression of BDNF signaling (via methylation, miRNAs, NRF2 interference), (**B**) synaptic gene misregulation (*DLG4*, *GRIN2B*, *SYN1*), (**C**) neurotransmitter imbalance (dopamine, glutamate), and (**D**) glial activation and neuroinflammation (e.g., ↑TNF-α, ↑IL-6). Long-term outcomes include (**Aa**) cognitive impairment and (**Ab**) transgenerational effects marked by altered chromatin states (e.g., H3K4me3, H3K9me2) and dysregulation of germline genes (*SYCP3*, *DAZL*), with consequences observable in F1 and F2 progeny. The figure is structured as a left-to-right flow from PFAS exposure to final outcomes. Arrows indicate the progression and direction of effects across molecular and cellular levels. This figure was created using BioRender.com.

To synthesize the mechanisms described in all of the sections above, Figure 1 illustrates the key molecular pathways through which PFAS disrupt neurodevelopment and epigenetic regulation across generations.

## 6. Conclusions

Current evidence paints a concerning picture of PFAS-induced neurotoxicity as a multifaceted process driven by interconnected epigenetic, genotoxic, and ncRNA-mediated mechanisms. Disruptions in DNA methylation and histone modifications—mediated through altered activity of key enzymes, such as DNMTs, TETs, HDACs, and methylation readers—can destabilize chromatin architecture and misregulate gene expression across critical neurodevelopmental periods. In parallel, the dysregulation of non-coding RNAs contributes to the silencing of neurotrophic, inflammatory, and synaptic genes critical for brain development and function.

Compounding these effects, PFAS exposure induces oxidative DNA damage and impairs repair mechanisms, leading to cumulative genomic instability that may further destabilize neuronal homeostasis. Although most findings are derived from in vitro and animal models, growing evidence suggests that these molecular disturbances are not confined to directly exposed individuals. Instead, heritable alterations in gene expression and germline epigenetic reprogramming are increasingly observed in the offspring of exposed organisms.

Although brain-specific transgenerational data remain limited, the persistent dysregulation of neurodevelopmental gene networks in exposed progeny suggests that PFAS-induced epigenetic reprogramming may have enduring consequences for brain health. This underscores the urgent need for future research that integrates cell-type-specific epigenome mapping, detailed chromatin conformation studies, and multi-generational analysis of neural tissues.

Clarifying these mechanisms will not only deepen our understanding of PFAS-related neurotoxicity but also help identify early biomarkers of exposure and guide the development of target interventions to protect vulnerable populations. As previously discussed, emerging biomarkers include neurotrophic factors, such as BDNF, altered miRNA profiles (e.g., miR-124, miR-132), and oxidative stress markers (e.g., 8-OHdG), which have shown relevance in both animal models and human studies. PFOS exposure has been linked to BDNF downregulation and epigenetic silencing in neuronal cells through mechanisms involving promoter methylation, histone modification, and miRNA-mediated regulation, notably miR-22 and miR-16. In parallel, miRNA alterations associated with PFAS exposure have been identified in cord blood and linked to neurodevelopmental outcomes in children. These biomarkers are detectable in accessible tissues like blood or placenta, enhancing their feasibility for biomonitoring in both research and clinical settings.

It is important to note that most epigenetic and neurotoxic effects reported in experimental systems have been observed at PFAS concentrations ranging from 1 to 10 µM, levels substantially higher than those typically detected in the general human population, which range between 2 and 60 nM in serum or cord blood, according to the EFSA 2020 scientific opinion report. However, in vivo studies using zebrafish, amphibians, or rodents have shown that internal concentrations may reach the lower micromolar range, particularly following high or prolonged exposure. Moreover, PFAS are known to bioaccumulate in human tissues, including the brain, liver, and placenta, with long biological half-lives that can lead to steady-state tissue concentrations higher than those in plasma. While these findings suggest a potential for biological overlap, especially in highly exposed populations, they also highlight a translational gap. Thus, further studies incorporating internal dosimetry, tissue-specific PFAS accumulation, and physiologically based pharmacokinetic (PBPK) modeling are essential to evaluate the human relevance of mechanisms observed in experimental models.

As the field moves forward, the intersection of environmental exposure, epigenetic regulation, and brain development should remain a key focus for toxicological research, biomarker discovery, and public health policy. Validating molecular signatures in longitudinal human studies will be critical to support early screening tools and refine regulatory thresholds for neurodevelopmental protection.

## Data Availability

No new data were created or analyzed in this study.
